# Patients with Fuchs Endothelial Dystrophy and Cataract Undergoing Descemet Stripping Automated Endothelial Keratoplasty and Phacoemulsification with Intraocular Lens Implant: Staged versus Combined Procedure Outcomes

**DOI:** 10.1155/2015/172075

**Published:** 2015-05-12

**Authors:** Evripidis Sykakis, Fook Chang Lam, Panagiotis Georgoudis, Samer Hamada, Damian Lake

**Affiliations:** Corneoplastic Unit and Eye Bank, Queen Victoria Hospital, East Grinstead RH19 3DZ, UK

## Abstract

*Purpose*. To compare the surgical outcomes of staged and combined phacoemulsification with intraocular lens implant (phaco+IOL) and Descemet stripping automated endothelial keratoplasty (DSAEK) in patients with Fuchs' endothelial dystrophy and cataract. *Setting*. Corneoplastic Unit and Eye Bank, Queen Victoria Hospital, East Grinstead, UK. *Methods*. Retrospective study of patients who had combined phaco+IOL and DSAEK (group 1) or phaco+IOL followed within 2 months by DSAEK (group 2). Patients who had previous eye surgery or any other ocular comorbidities were excluded. *Results*. There were 28 eyes in group 1 and 31 in group 2. There were no significant differences in the demographics and corneal tissue characteristics of the two groups. The endothelial disc dislocation and rebubbling rate within 1 week in group 1 was 21.42% and in group 2 was 3.2% (*P* = 0.04), while the endothelial cell density at 12 months was 1510 ± 433 for group 1 and 1535 ± 482 for group 2 (*P* = 0.89). The mean 12-month logMAR visual acuity was 0.28 ± 0.24 for group 1 and 0.33 ± 0.15 for group 2 (*P* = 0.38). *Conclusions*. Although the combined procedure seems to be associated with a higher complication rate the final outcomes seem to be similar to both methods.

## 1. Introduction

Endothelial keratoplasty (EK) in the forms of Descemet stripping automated endothelial keratoplasty (DSAEK) and Descemet membrane endothelial keratoplasty (DMEK) has revolutionised corneal transplantation for the treatment of endothelial dysfunction and is the procedure of choice in such cases, as it carries several advantages such as faster rehabilitation, better refractive outcomes, no suture related problems, smaller risk of traumatic graft dehiscence, and less risk of corneal graft rejection [1–4]. Although DMEK has gained interest over the last few years due to improved visual acuity results and decreased rejection rates, the associated technical challenges have limited widespread acceptance and DSAEK still remains the most common endothelial keratoplasty procedure [5].

In patients with Fuchs endothelial corneal dystrophy (FECD) and cataract that require both DSAEK and phacoemulsification plus intraocular lens implantation (IOL), there is a difference of opinion as to whether the surgery should be performed concurrently or sequentially with the DSAEK following shortly after the phacoemulsification plus IOL implantation. Some authors have advocated that a staged procedure should be preferred [3] while others have presented supporting evidence for the combined new triple procedure [6, 7].

In this study, we are presenting the results of a retrospective comparative study performed in a UK tertiary referral corneal unit where every effort has been made to perform a direct comparison of the 2 surgical options by making sure that there are no clinical or donor tissue related confounding factors.

## 2. Methods

Case notes review of all patients who had phacoemulsification followed by DSAEK within 2 months or phacoemulsification and DSAEK performed concurrently in our unit from January 2009 till December 2013 was performed. Only cases with documented diagnosis of FECD (with clinically evident stromal oedema and central guttata) and cataract (visually significant cataract or mild cataract with expectation of progression) and that completed at least 6-month follow-up were included. Exclusion criteria were other comorbidities like glaucoma, optic nerve or retinal disease age related macular degeneration, and previous ocular surgery. Only cases performed by the same senior surgeon (DL) were included. Which surgical approach was chosen in each case was dictated by clinical circumstances and the choice of either surgical option was not randomized.

Data collected and compared included patient demographics, tissue related parameters taken from our eye bank, and clinical data including pre- and postoperative best spectacle-corrected visual acuity (BSCVA), endothelial cell density (ECD), complication rates, and graft rejection or failure episodes. Data were compared at 6 and 12 months after surgery.

### 2.1. Surgical Technique

All cases were performed under Subtenon's or Peribulbar anaesthesia except in staged procedures where phacoemulsification and IOL implantation was performed under topical anaesthesia.

The cataract procedure was performed using the Infiniti phacoemulsification system (Alcon Inc., TE, USA) with the OZil Torsional Handpiece (Alcon Inc., TE, USA) and using a bimanual technique and a 2.2 mm sutureless main incision. The ophthalmic viscoelastic device (OVD) used in combined procedures was Healon GV (Abbott Medical Optics, IL, USA) while there was no preference regarding the OVD in cases where only cataract was performed. The preloaded one piece aspheric hydrophobic Tecnis ZCB00 (Abbott Medical Optics, IL, USA) was used in all cases.

In combined cases, the main incision was enlarged, while in DSAEK only procedures a 5.5 mm peripheral corneal incision was created. The stripping of Descemet's membrane was performed before removal of OVD with bimanual automated irrigation/aspiration in combined cases while in DSAEK only cases it was performed under air. OVD was thickly coated onto a Sheets glide and the endothelial graft placed endothelium down onto the OVD and then was transported to the corneal wound. The graft was delivered into the anterior chamber with a bent insulin syringe using a “push” technique. The wound was sutured with 10/0 nylon and anterior chamber (AC) reformed with BSS plus. Air was injected under the graft and the graft was centred. Following 100% air fill for 10 minutes, air was released to make a bubble just to the size of corneal graft button. At this stage, mydriatics were instilled and the patient was transferred to the ward to rest in a supine position. Intraocular pressure was checked at 1 hour postoperatively and air was released if above 30 mm Hg.

In both groups, the postoperative regime was G. Cyclopentolate 1% tds for 2 days, G. Chloramphenicol qds for 2 weeks, and G. Dexamethasone 0.1% every 2 hours, tapering dose over 6 months.

The tissue used was processed by Queen Victoria Eye Bank and was precut by the same eye bank technician using the disposable Horizon microkeratome after being mounted on the system's plastic artificial chamber with pressurized air (Refractive Technologies, Cleveland, OH). A new disposable microkeratome was used for each donor cornea. The microkeratome head was selected after pachymetry was performed with a Sonogage Horizon System (Refractive Technologies, Cleveland, OH), aiming for a 100 *μ*m thickness endothelial graft. All grafts were prepared in a sterile clean room facility licensed by the Human Tissue Authority and transported to the operating room in an Optical chamber (Independent Corneal Viewing Chamber, Bausch & Lomb, St. Louis, MO).

The surgeon trephined the tissue in theatre with the Iowa press system and placed the graft in BSS plus whilst preparing the recipient eye. The size of the donor corneal disc ranged from 8.5 to 9 mm in diameter.

### 2.2. Specular Microscopy

Endothelial cell density following surgery was measured using a noncontact specular microscope (EM-3000, Tomey, USA) at the centre of the cornea. Specular images of more than 60 cells were analysed.

### 2.3. Statistical Analysis

All statistical analyses were performed with SPSS for Windows version 17.0 (PASW, USA). Mann-Whitney* U* test, Wilcoxon signed rank test, and Fisher's exact test were used. All data are expressed as mean ± SD. A level of *P* < 0.05 was accepted as statistically significant.

## 3. Results

Out of the total 202 DSAEK operations performed in this period of time in our unit, only 59 cases fulfilled the inclusion and exclusion criteria. Out of these 59 cases, 28 eyes had a combined (group 1) and 31 eyes had a staged (group 2) procedure performed.

### 3.1. Patient Demographics and Donor Tissue Characteristics

The average age of the overall group of patients (*n* = 59) was 73.6 ± 9.31 years (range, 57–91 years), with 60.3% women and 39.7% men. The average age of the patients with staged procedure (*n* = 31) was 76.74 ± 8.9 years (range, 62–91 years) compared with an average age of 70.15 ± 8.54 years (range, 57–90 years) for patients receiving DSAEK combined with cataract surgery (*n* = 28).

The donor tissue characteristics and quality used for patients in the two groups were the same and there was no clinical or statistical significant difference between endothelial cell count, donor age and tissue retrieving, storage, and preparation factors. All these data are presented in detail in Table 1.

### 3.2. Clinical Outcomes

All cases had 6-month follow-up data available while 26 cases from group 1 (92.8%) and 29 cases from group 2 (93.5%) had 12-month follow-up data available.

The preoperative logMAR BSCVA was 0.70 ± 0.44 for group 1 and 0.82 ± 0.40 for group 2 (*P* = 0.26). BSCVA at 6 and 12 months and ECD at 6 and 12 months are presented in Table 2. The percentage of ECD loss at 12 months was 45.3% and 46.2% for group 1 and 2, respectively (*P* = 0.95). There was no iatrogenic primary graft failure and no rejection episodes occurred 12 months following surgery.

Fluid interface between recipient cornea and donor disc as well as partial or complete donor disc dislocation within the first week was observed in 6 cases in group 1 (21.42%) and in 1 case in group 2 (3.2%) (*P* = 0.04). Two cases from group 1 had to be taken into theatre for graft repositioning and air injection (7.14%) while all the other cases settled following air injection in the anterior chamber and posturing, performed as an office procedure. Despite further manipulations performed, clinical outcome was successful in all 7 cases.

## 4. Discussion

There has been lots of discussion within the ophthalmology community regarding whether to do a staged or triple simultaneous procedure for patients with FECD and cataracts. Some authors have advocated that a staged procedure should be preferred [3] while others have presented supporting evidence for the combined triple procedure [6, 7]. Up to date, there is lack of enough published evidence as to which should be the preferred method. There have been no randomized clinical trials and the only study actually comparing the outcomes of the two methods is the early study by Terry et al. [7]. In the specific paper, the authors, following a large prospective case series of 315 eyes that had DSAEK, performed a retrospective analysis of patients that underwent a staged or combined procedure and, among other results, presented and compared a cohort of 25 DSAEKs without other major comorbidities and 75 triple procedures. Their conclusion was that both methods were equally effective with similar endothelial mean cell loss in both groups with 33% in the staged versus 32% in the combined procedure, which compared better to our 46.2% versus 45.3%.

Although the above authors had a significantly larger group of patients, there seems to be a lot of heterogeneity in the background clinical history as well as the donor tissue used. Additionally, the triple procedure group was 3-fold bigger than the DSAEK one.

In our study, we have performed a direct comparison of the 2 surgical options by making sure that there are no clinical or donor tissue related confounding factors.

Looking into the donor disc dislocation rates in the literature, it is difficult to make direct comparisons as different studies report dislocation rates differently. Dislocations may represent either fluid in the interface of an otherwise well-positioned graft or complete dislocation into the anterior chamber [8]. We have decided to include both in our results. Thus reported dislocation rates in the literature range from 2.5% [7] to 14% [8] while in our 59 cases dislocation rate was 11.8%. Although no significant difference as far as graft dislocations was found in the study by Terry et al. [7], in our comparison, there is a statistically and clinically significant difference between the two groups and, by our results, it looks like it is 6 times more likely to develop dislocation or fluid interface with the combined technique. Many surgeons believe that the use of OVD during descematorhexis is a risk factor for graft dislocation [9], something not proven in Terry's study as OVD was used in all their cases. The difference between the OVDs used in the 2 studies (Healon GV in our cohort versus Healon in Terry et al.) could be a contributing factor as the OVD used by us has greater viscosity. Although we prefer the use of Healon GV for easier visualisation during removal, one could argue that there could still be remnants of OVD that could possibly contribute to that difference in dislocation rate. Nevertheless, one point to be taken into consideration by surgeons performing the procedures is the importance of choosing the appropriate OVD or perhaps performing descematorhexis under air or under BSS with the use of an AC maintainer.

Other reasons for this difference in the dislocation rate could be that in the staged procedure the position of the IOL is far more stable in the capsular bag making the surgery more straightforward or the fact that when performing the DSAEK alone, there is lower vitreous pressure, making it easier to insert, unfold, and manipulate the graft.

There are definite limitations in our study as it is a retrospective one where no randomisation has been performed regarding procedure choice and a prospective randomised trial would definitely give more accurate information on the* pro et contra* of each approach, but due to the lack of efficient evidence in the current literature, we believe that useful conclusions can be drawn from our comparison.

The results of our study show that although the complication rate may be higher in the case of combined procedures, the final outcomes are equally good regarding final visual acuity and endothelial cell count. Although this is evidence that maybe the staged approach should be the preferred method, one could argue that the combined approach is still more cost effective and convenient as by choosing it the patient will only need a secondary procedure in the operating theatre in only 21.4% of the cases while with the staged approach 2 operations are* de facto* needed.

## Figures and Tables

**Table 1:** 

Donor tissue characteristics	Group 1	Group 2	*P* value
Donor age	63.06 ± 14.42	60.28 ± 15.02	0.46
Donor endothelial cell density	2766 ± 147	2803 ± 160	0.35
Death to retrieval time (hours)	14.9 ± 6.5	16.61 ± 6.34	0.53
Retrieval to storage time (hours)	5.8 ± 3	4.6 ± 5.9	0.59
Storage before preparation time (days)	6 ± 2.8	8 ± 6.5	0.47
Preparation of donor disc prior to surgery time (hours)	4.7 ± 1.9	5.1 ± 2.7	0.72

Group 1: combined procedure; Group 2: staged procedure.

**Table 2:** 

BSCVA and ECD at 6 and 12 months	Group 1	Group 2	*P* value
BSCVA at 6 months	0.4 ± 0.32	0.49 ± 0.24	0.23
BSCVA at 12 months	0.28 ± 0.24	0.33 ± 0.15	0.38
ECD at 6 months	1584 ± 364	1588 ± 299	0.97
ECD at 12 months	1510 ± 433	1535 ± 482	0.89

Group 1: combined procedure; Group 2: staged procedure.
